# Fibroblast Growth Factor 19 in Gestational Diabetes Mellitus and Fetal Growth

**DOI:** 10.3389/fendo.2021.805722

**Published:** 2022-01-25

**Authors:** Meng-Nan Yang, Rong Huang, Xin Liu, Ya-Jie Xu, Wen-Juan Wang, Hua He, Guang-Hui Zhang, Tao Zheng, Fang Fang, Jian-Gao Fan, Fei Li, Jun Zhang, Jiong Li, Fengxiu Ouyang, Zhong-Cheng Luo

**Affiliations:** ^1^ Ministry of Education-Shanghai Key Laboratory of Children’s Environmental Health, Early Life Health Institute, and Department of Pediatrics, Xinhua Hospital, Shanghai Jiao-Tong University School of Medicine, Shanghai, China; ^2^ Department of Obstetrics and Gynecology, Lunenfeld-Tanenbaum Research Institute, Prosserman Centre for Population Health Research, Mount Sinai Hospital, Institute of Health Policy, Management and Evaluation, Dalla Lana School of Public Health, Faculty of Medicine, University of Toronto, Toronto, ON, Canada; ^3^ Department of Clinical Assay Laboratory, Xinhua Hospital, Shanghai Jiao-Tong University School of Medicine, Shanghai, China; ^4^ Department of Obstetrics and Gynecology, Xinhua Hospital, Shanghai Jiao-Tong University School of Medicine, Shanghai, China; ^5^ Center for Fatty Liver, Shanghai Key Lab of Pediatric Gastroenterology and Nutrition, Department of Gastroenterology, Xinhua Hospital, Shanghai Jiao Tong University School of Medicine, Shanghai, China; ^6^ Department of Clinical Medicine-Department of Clinical Epidemiology, Aarhus University Hospital, Aarhus, Denmark

**Keywords:** fibroblast growth factor 19 (FGF19), gestational diabetes (GD), fetal growth, insulin, C-peptide

## Abstract

Fibroblast growth factor 19 (FGF19) has been implicated in glucose homeostasis. Gestational diabetes mellitus (GDM) enhances fetal insulin secretion and fetal growth. Girls weigh less and are more insulin resistant than boys at birth. We sought to assess whether FGF19 is associated with GDM and fetal growth and explore potential sex dimorphic associations. This was a nested case-control study in the Shanghai Birth Cohort, including 153 pairs of newborns of GDM versus euglycemic mothers matched by infant’s sex and gestational age at birth. Cord plasma FGF19, insulin, C-peptide, proinsulin, IGF-I and IGF-II concentrations were measured. Cord plasma FGF19 concentrations were similar in GDM versus euglycemic pregnancies (mean ± SD: 43.5 ± 28.2 versus 44.5 ± 30.2 pg/mL, P=0.38). FGF19 was not correlated with IGF-I or IGF-II. FGF19 concentrations were positively correlated with birth weight (r=0.23, P=0.01) and length (r=0.21, P=0.02) z scores, C-peptide (r=0.27, P=0.002) and proinsulin (r=0.27, P=0.002) concentrations in females. Each SD increment in cord plasma FGF19 was associated with a 0.25 (0.07-0.43) increase in birth weight z score in females. In contrast, FGF19 was not correlated with birth weight or length in males. These sex dimorphic associations remained after adjusting for maternal and neonatal characteristics. The study is the first to demonstrate that GDM does not matter for cord blood FGF19 concentrations. The female specific positive correlation between FGF19 and birth weight is suggestive of a sex-dimorphic role of FGF19 in fetal growth. The observations call for more studies to validate the novel findings and elucidate the underlying mechanisms.

## Introduction

Fibroblast growth factor 19 (FGF19) is mainly secreted from the enterocytes in the distal part of the small intestine (1), and is expressed in fetal brain, cartilage, skin and retina (2, 3). FGF19 has been implicated in the regulation of glucose and lipid metabolism (4–6). FGF19 may regulate glucose homeostasis *via* enhancement of glycogen synthesis, glucose catabolism and suppression of gluconeogenesis (5, 7, 8). Decreased circulating FGF19 levels have been reported in obese, insulin resistant and type 2 diabetes patients (9–11). Animal studies suggest that FGF19 may be negatively correlated with adiposity and positively correlated with insulin sensitivity (12–16), and may even reverse diabetes (17, 18).

Gestational diabetes mellitus (GDM) (*de novo* glucose intolerance in pregnancy) is a common cause of enhanced fetal insulin secretion and overgrowth (19), and has been associated with reduced maternal circulating FGF19 levels (20) and placental FGF19 expression (21), and reduced fetal insulin sensitivity (22). We thus hypothesize that GDM may affect fetal FGF19 levels. However, there have been no studies on whether GDM may affect FGF19 concentrations in fetuses or newborns. FGF19 may induce glycogen and protein synthesis in the liver (7, 23) and skeletal muscle mass (24). However, data are scarce concerning whether FGF19 may affect fetal growth in humans. We are aware of only one small study (n=44) which did not detect any association (25). Girls weigh less and are more insulin resistant than boys at birth (26). It is unknown whether any association between FGF19 and fetal growth may differ by sex. Considering theses research data gaps, we sought to examine whether cord blood FGF19 is associated with GDM and fetal growth and explore potential sex-dimorphic associations. The primary research questions are whether GDM affects cord blood FGF19 concentrations, and whether there are differential correlations between FGF19 and fetal growth in males and females.

## Methods

### Study Design, Subjects and Specimens

We conducted a nested matched (1:1) case-control study in the Shanghai Birth Cohort (SBC) (27). The SBC is a large, carefully phenotyped birth cohort with linked biospecimen bank for studies on perinatal determinants of infant growth, development and health. Women at preconception or early pregnancy care clinics were recruited from six university-affiliated tertiary obstetric care hospitals in Shanghai between 2013 and 2016, including a total of 4127 pregnancies. The women were followed up at the first, second and third trimesters of pregnancy and delivery. Data and specimens were collected at each study visit. All collected blood samples were kept on ice, stored temporarily in a 4°C refrigerator and centrifuged within 2 hours after the specimen collection. The separated serum and EDTA plasma samples were stored in multiple aliquots at -80°C until assays. The study was approved by the research ethics committees of Xinhua Hospital (the coordination center, reference number M2013-010) and all participating hospitals. Written informed consent was obtained from all study participants.

GDM was diagnosed according to the International Association of Diabetes and Pregnancy Study Groups (IADPSG) criteria (28) - if any one of the blood glucose values was at or above the following thresholds in the 75 g oral glucose tolerance test (OGTT) at 24-28 weeks of gestation: fasting 5.1 mmol/L, 1-hour 10.0 mmol/L and 2-hour 8.5 mmol/L.

As part of the SBC, a nested case control study was designed to study the impacts of GDM on metabolic health biomarkers in the newborns (29). Briefly, cases were the newborns of GDM mothers (n=153), and controls were the newborns of euglycemic mothers (n=153) in the SBC. Cases and controls were matched (1:1) by infant’s sex (the same) and gestational age at birth (within 1 week). The present study reported FGF19 in association with GDM and fetal growth.

Birth weight z scores were calculated using the 2015 Chinese sex- and gestational age-specific birth weight standards (30). Birth length z scores were calculated according to sex- and gestational age-specific means and SDs of all singleton infants in the SBC.

### Biochemical Assays

Cord plasma FGF19 was measured by an enzyme-linked immunosorbent assay (ELISA) kit (R&D system, Minnesota, USA), and the absorbance was determined using a microplate spectrophotometer (Beckman CX7, USA). Serum insulin and insulin-like growth factor I (IGF-I) concentrations were detected by chemiluminescent assays (ADVIA Centaur and Immulite 2000, Siemens, Germany). Plasma IGF-II was measured by an ELISA kit from R&D systems (Minnesota, USA). Plasma C-peptide and proinsulin were measured by ELISA kits from Mercodia system (Uppsala, Sweden). Plasma total and high-molecular-weight (HMW) adiponectin were measured by an ELISA kit from ALPCO (Salem, NH, USA). Plasma leptin was measured by an ELISA kit from Invitrogen (Carlsbad, CA, USA). The detection limits were 1.17 pg/mL for FGF19, 3.5 pmol/L for insulin, 25 ng/mL for IGF-I, 1.88 pg/mL for IGF-II, 25 pmol/L for C-peptide, and 1.7 pmol/L for proinsulin, 0.034 ng/mL for HMW and total adiponectin, and 3.5 pg/mL for leptin, respectively. Intra-assay and inter-assay coefficients of variation were in the ranges of 6.4-11.6% for FGF19, 2.0-6.5% for insulin and IGF-I, 5.0-8.6% for proinsulin, 0.4-13.5% for C-peptide, 2.4-9.3% for IGF-II, and 6.9%-10.4% for leptin, HMW and total adiponectin, respectively. In all biomarker assays, the laboratory technicians were blinded to the clinical status (GDM or not) of study subjects.

### Statistical Analysis

Data are presented as mean ± SD (standard deviation) or median (interquartile range) for continuous variables, and frequency (percentage) for categorical variables. Log-transformed biomarker data were used in t-tests, correlation and regression analyses. Pearson partial correlation was used to evaluate the correlations with biomarkers adjusting for gestational age at cord blood sampling/delivery. Differences in correlations in male and female infants were examined by Fisher’s z test. Generalized linear models were applied to assess the associations of cord blood FGF19 with GDM, fetal growth and fetal growth factors adjusting for maternal and neonatal characteristics. The co-variables included maternal age, ethnicity, parity, education (university: yes/no), pre-pregnancy BMI (kg/m^2^), smoking or alcohol use in pregnancy (yes/no), gestational hypertension, family history of diabetes, family history of hypertension, mode of delivery (caesarean section/vaginal), infant’s sex and gestational age (weeks) at birth. We estimated the changes in birth weight and length z scores per SD increment in FGF19. Mediation analyses were conducted to assess whether fetal growth factors may mediate any associations between FGF19 and fetal growth (birth weight or length) using the product (“Baron and Kenney”) method (31). Data management and analyses were performed in SAS version 9.4 (SAS Institute, Cary, NC). Two-tailed P<0.05 was considered statistically significant in evaluating the primary research question on whether cord plasma FGF19 concentrations differ between GDM and controls, or whether there are differential correlations between FGF19 and fetal growth (birth weight z score as the primary indicator) in males and females.

## Results

### Maternal and Neonatal Characteristics

Characteristics of study subjects in this nested study in the Shanghai birth cohort have been described recently (29). Briefly, women with GDM had higher pre-pregnancy BMI than euglycemic women (Mean: 23.6 versus 21.6 kg/m^2^) and were more likely to have gestational hypertension (5.2% versus 0.6%) and tended to be more likely to have a family history of diabetes (16.5% versus 9.5%) than women with a euglycemic pregnancy. There were no significant differences in other maternal characteristics including maternal age, education, parity, alcohol use or smoking during pregnancy. The newborns of GDM mothers were more likely to be delivered by caesarean section than the newborns of euglycemic mothers (54.3% versus 33.1%). Mean gestational age at delivery was about 39 weeks in both GDM and euglycemic pregnancies (39.1 and 39.3 weeks). There were 142 (46.6%) caesarean section deliveries (97 elective caesarean sections), and 13 (4.3%) preterm births (<37 weeks in gestational age, all between 34-36 weeks).

### Cord Blood FGF19 and Fetal Growth Factors

Adjusting for maternal and neonatal characteristics including pre-pregnancy BMI, family history of hypertension, parity, cesarean section and gestational age at delivery (other covariates did not affect the comparisons, all P>0.2), cord plasma FGF19 (43.5 ± 28.2 versus 44.5 ± 30.2 pg/mL, P=0.38), proinsulin (22.3 ± 17.9 versus 19.3 ± 16.8 pmol/L, P=0.91) and C-peptide (264.3 ± 185.7 versus 269.3 ± 155.3 pmol/L, P=0.29) concentrations were similar in GDM and euglycemic pregnancies (**Table 1**).

**Table 1:** Cord plasma FGF19, proinsulin and C-peptide concentrations in the newborns of GDM versus euglycemic (control) mothers (matched by infant sex and gestational age) in the Shanghai Birth Cohort.

	GDM (n=153)	Control (n=153)	Crude P*	Adjusted P*
FGF19	43.5±28.2	44.5±30.2	0.88	0.38
(pg/mL)	35.2 (25.5, 53.8)	36.0 (24.1, 52.5)		
Proinsulin	22.3±17.9	19.3±16.8	0.66	0.91
(pmol/L)	16.5 (11. 5, 27.2)	15.6 (11.3, 21.4)		
C-Peptide	264.3±185.7	269.3±155.3	0.09	0.29
(pmol/L)	230.0 (141.0, 344.0)	243.4 (162.6, 339.2)		

Data presented are mean±SD and median (inter-quartile range).

GDM, Gestational diabetes mellitus; FGF19, Fibroblast growth factor 19.

*Crude P values were from paired t-tests. Adjusted P values were from generalized linear models in the comparisons of log-transformed biomarker data between the two groups adjusted for maternal (pre-pregnancy BMI, family history of hypertension, parity) and neonatal (cesarean section, gestational age) characteristics; other factors (all P>0.2) were excluded since they were similar and did not affect the comparisons. All comparisons were based on log-transformed data for biomarkers.

Descriptive data on cord plasma insulin, IGF-I, IGF-II, leptin, total and HMW adiponectin were reported elsewhere (29, 32). Briefly, cord plasma IGF-I (76.6 ± 27.8 versus 68.1 ± 25.1 ng/mL, P=0.008) and IGF-II (195.3 ± 32.5 versus 187.5 ± 30.8 ng/mL, P=0.04) concentrations were significantly higher in GDM versus euglycemic pregnancies, while insulin concentrations were not significantly different (29). Cord plasma HMW adiponectin concentrations were lower in GDM versus euglycemic pregnancies, while leptin and total adiponectin concentrations were not significantly different (32).

### Determinants of Cord Blood FGF19

Cord plasma FGF19 concentrations were similar in male versus female newborns (43.9 ± 29.4 versus 44.2 ± 29.1 pg/mL, P=0.46), and were higher in preterm versus term births (64.6 ± 33.4 versus 43.2 ± 28.8 pg/mL, P=0.009) and in caesarean section versus vaginal deliveries (47.1 ± 28.7 versus 41.4 ± 29.5 pg/mL, P=0.09). Compared with vaginal deliveries, FGF19 concentrations were higher in elective caesarean sections (49.9 ± 30.3 pg/mL, P=0.03), but similar in emergency caesarean sections (40.9 ± 24.2 pg/mL, P=0.92). Other maternal and neonatal factors were not associated with cord plasma FGF19 concentrations (all P>0.05).

### FGF19 in Correlations With Fetal Growth and Fetal Growth Factors

**Figure 1:**
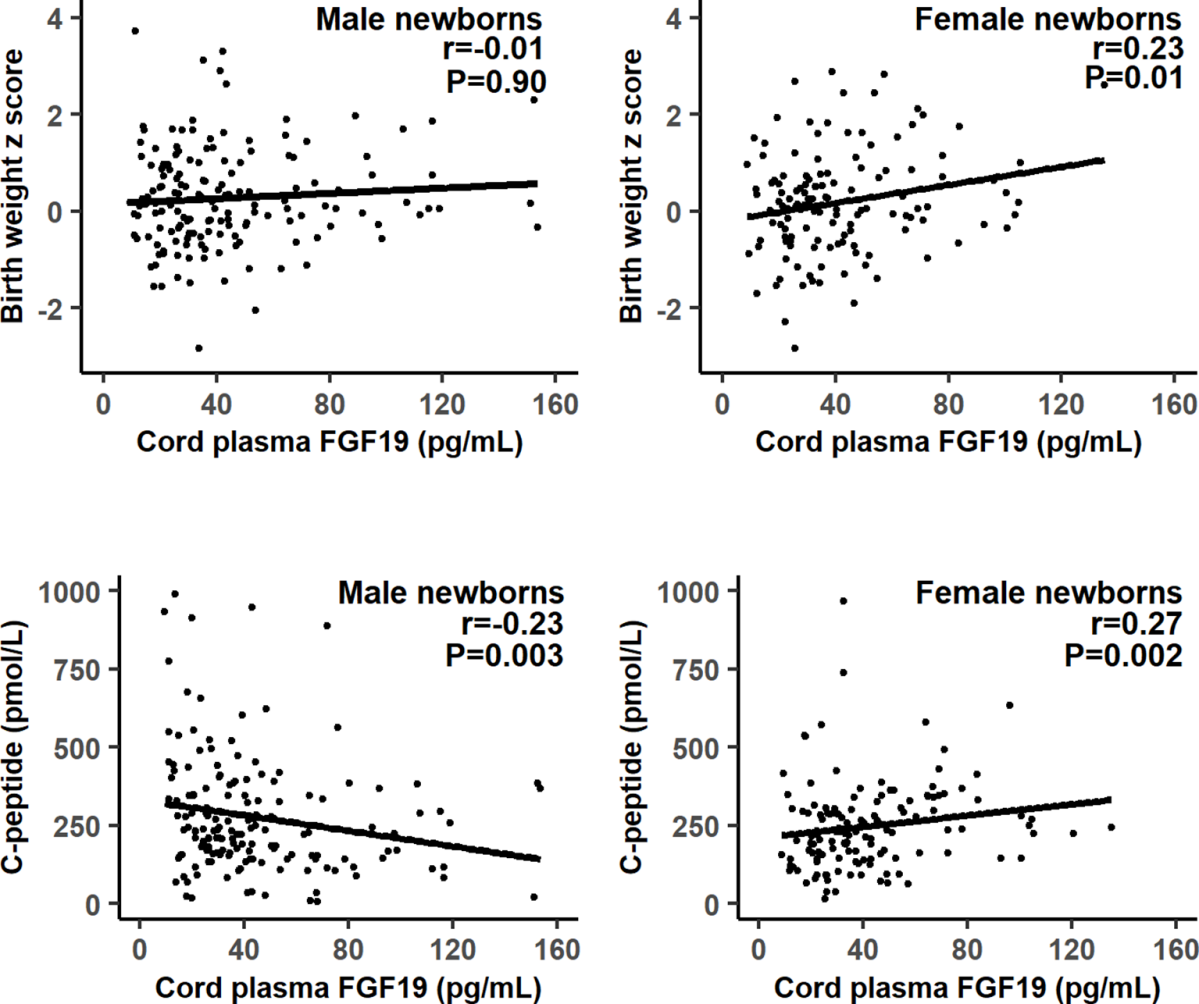
Scatter plots illustrating the differential correlations between cord plasma fibroblast growth factor 19 (FGF19) and fetal growth (birth weight z score) or C-peptide in males and females.

There were significant interactions (all P<0.05) between FGF19 and infant sex in relation to fetal growth (birth weight or length z score), C-peptide and proinsulin. Therefore, we present the correlation coefficients stratified by infant’s sex. Gestational age was negatively correlated with cord plasma FGF19 (r=-0.17, P=0.003). Adjusting for gestational age at delivery/blood sampling, cord plasma FGF19 concentrations were positively correlated with birth weight (r=0.23, P=0.01) and birth length (r=0.21, P=0.02) z scores, C-peptide (r=0.27, P=0.002) and proinsulin (r=0.27, P=0.002) concentrations in female newborns (**Table 2** and **Figure 1**). The positive correlation between cord plasma FGF19 and birth weight in females remained if further adjusting for cord plasma C-peptide (r=0.21, P=0.01). In contrast, cord plasma FGF19 was not correlated with birth weight or length but was negatively correlated with C-peptide (r=-0.23, P=0.003) in male newborns. The correlations between FGF19 and proinsulin or C-peptide are in opposite directions in females and males (tests for differences in correlation coefficients, all P<0.05). Cord plasma FGF19 was not correlated with IGF-I, IGF-II, leptin, HMW or total adiponectin in both sexes. All these correlations were not significantly different in GDM and controls (**Table 3**).

**Table 2:** Cord blood FGF19 in correlations with birth weight, birth length, fetal growth factors, leptin and adiponectin in males and females.

	Males		Females		
	r	P	r	P	P*
Birth weight	-0.01	0.90	0.23	**0.01**	**0.039**
Birth length	-0.04	0.66	0.21	**0.02**	**0.045**
IGF-I	0.01	0.90	0.02	0.79	0.91
IGF-II	0.07	0.37	0.05	0.54	0.89
Proinsulin	-0.13	0.10	0.27	**0.002**	**<0.001**
Insulin	0.02	0.83	0.15	0.09	0.26
C-peptide	-0.23	**0.003**	0.27	**0.002**	**<0.001**
Leptin	-0.14	0.08	0.11	0.22	0.12
Adiponectin, HMW	-0.01	0.87	-0.002	0.98	0.91
Adiponectin, Total	0.05	0.49	0.04	0.64	0.88

Data presented are Pearson partial correlation coefficients adjusting for gestational age at delivery/cord blood sampling. Data were in z scores for birth weight and length, and were log-transformed for biomarkers in the correlation analyses.

FGF19, Fibroblast growth factor 19; IGF-I, insulin-like growth factor-I; IGF-II, insulin-like growth factor-II; HMW, high molecular weight.

*P values in z tests for differences in correlation coefficients in males and females.

P values in bold: P < 0.05.

**Table 3:** Cord blood FGF19 in correlations with birth weight, birth length and fetal growth factors, leptin and adiponectin in the newborns of GDM and euglycemic (control) mothers.

	GDM		Control		
	r	P	r	P	P*
Birth weight	0.13	0.12	0.07	0.43	0.57
Birth length	0.18	**0.035**	-0.02	0.86	0.11
IGF-I	0.13	0.13	-0.07	0.41	0.10
IGF-II	0.15	0.07	-0.02	0.82	0.15
Proinsulin	0.10	0.23	-0.03	0.67	0.25
Insulin	0.11	0.20	0.03	0.70	0.52
C-peptide	-0.0004	1.00	-0.08	0.33	0.49
Leptin	0.04	0.63	-0.11	0.17	0.09
Adiponectin, HMW	-0.07	0.42	0.06	0.45	0.41
Adiponectin, Total	0.03	0.75	0.08	0.35	0.92

Data presented are Pearson partial correlation coefficients adjusting for gestational age at delivery/cord blood sampling. Data were in z scores for birth weight and length, and were log-transformed for biomarkers in the correlation analyses.

FGF19, Fibroblast growth factor 19; IGF-I, insulin-like growth factor-I; IGF-II, insulin-like growth factor-II; HMW, high molecular weight.

*P values in comparisons of correlation coefficients in GDM and control infants.

P values in bold: P < 0.05.

### Adjusted Associations Between FGF19 and Fetal Growth

Adjusting for maternal and neonatal characteristics in female newborns, each SD increment in cord plasma FGF19 was associated with a 0.25 [95% confidence interval (CI): 0.07, 0.43] increase in birth weight (P=0.01), a 0.20 (0.01, 0.40) increase in birth length (P=0.047), a 0.18 (0.09, 0.27) increase in cord blood insulin (P<0.001), a 0.32 (0.17, 0.47) increase in C-peptide (P<0.001), and a 0.44 (0.24, 0.64) increase in proinsulin (P<0.001), respectively (**Table 4**, standardized regression coefficients; all continuous variables in z scores in the regression analyses). In contrast, in male newborns, cord plasma FGF19 was not associated with birth weight, length, insulin or proinsulin, while each SD increment in FGF19 was associated with a 0.19 (0.02, 0.37) decrease in C-peptide (P=0.03) (opposite to the association in female newborns).

**Table 4:** The adjusted associations of cord blood FGF19 with birth weight, birth length, insulin, proinsulin, C-peptide, IGF-I and IGF-II, leptin and adiponectin in males and females.

Data in z score	Males β (95% CI)	P	Females β (95% CI)	P
Birth weight	-0.07 (-0.25, 0.10)	0.40	0.25 (0.07, 0.43)	**0.008**
Birth length	-0.06 (-0.24, 0.13)	0.54	0.20 (0.01, 0.40)	**0.047**
Insulin	0.16 (-0.04, 0.37)	0.12	0.18 (0.09, 0.27)	**<0.001**
Proinsulin	-0.11 (-0.25, 0.04)	0.15	0.44 (0.24, 0.64)	**<0.001**
C-peptide	-0.19 (-0.37, -0.02)	**0.03**	0.32 (0.17, 0.47)	**<0.001**
IGF-I	-0.10 (-0.26, 0.06)	0.21	0.06 (-0.13, 0.26)	0.50
IGF-II	0.08 (-0.08, 0.23)	0.34	0.07 (-0.12, 0.27)	0.46
Leptin	-0.06 (-0.20, 0.08)	0.40	0.08 (-0.12, 0.27)	0.45
Adiponectin, HMW	0.01 (-0.15, 0.16)	0.93	-0.07 (-0.25, 0.11)	0.45
Adiponectin, Total	-0.02 (-0.19, 0.14)	0.78	-0.03 (-0.19, 0.14)	0.75

Data (β) presented are the standardized changes in birth weight, birth length or each fetal growth factor per SD increment in FGF19 from generalized linear models adjusting for maternal (pre-pregnancy BMI, family history of hypertension, parity) and neonatal (cesarean section, gestational age) characteristics; other maternal and neonatal factors were excluded since they did not affect the comparisons (all P>0.2). Standardized z score data were used for all continuous variables in the regression analyses. The SDs for calculating the z scores in cord blood biomarkers were 29.2 pg/mL for FGF19, 55.2 pmol/L for insulin, 17.4 pmol/L for proinsulin, 170.7 pmol/L for C-peptide, 26.8 ng/mL for IGF-I, and 31.8 ng/mL for IGF-II, 7.2 ng/mL for leptin, 7.8 (μg/mL) for HMW adiponectin and 15.4 (μg/mL) for total adiponectin, respectively.

There were significant interactions between FGF19 and sex in relation to birth weight (P=0.019), birth length (P=0.055), proinsulin (P < 0.0001) and C-peptide (P < 0.0001).

P values in bold: P < 0.05.

### Mediation Analyses

Mediation analyses showed that cord blood proinsulin could partly explain the association between FGF19 and fetal growth in female newborns (**Table 5**). Proinsulin could account for 34.8% of the effect size of FGF19 for birth weight (P=0.037), and 55.1% of the effect size for birth length (P=0.016), respectively. There were no significant mediation effects for other fetal growth factors (insulin, C-peptide, IGF-I or IGF-II) in the associations of FGF19 with birth weight and length.

**Table 5:** Mediation analyses in the associations of cord blood FGF19 with fetal growth (birth weight, birth length) in female newborns (n=140).

	Birth weight z score	P	Birth length z score	P
FGF19 (total effect)	0.25 (0.07, 0.43)	**0.008**	0.20 (0.01, 0.40)	**0.047**
*Mediation by				
Insulin	0.01 (-0.05, 0.08)	0.71	0.006 (-0.07, 0.08)	0.88
Proinsulin	0.09 (0.006, 0.17)	**0.037**	0.11 (0.02, 0.20)	**0.016**
C-peptide	-0.02 (-0.09, 0.05)	0.61	-0.02 (-0.10, 0.06)	0.65
IGF-I	0.02 (-0.05, 0.10)	0.51	0.01 (-0.02, 0.04)	0.55
IGF-II	0.02 (-0.03, 0.07)	0.47	0.01 (-0.02, 0.05)	0.49

Data (β) presented are the changes in fetal growth (birth weight or birth length) per SD increment in each cord blood biomarker from generalized linear models. Standardized z score data were used for all continuous predictor variables in the regression models. The SDs for calculating the z scores in cord blood biomarkers were 29.2 pg/mL for FGF19, 55.2 pmol/L for insulin, 17.4 pmol/L for proinsulin, 170.7 pmol/L for C-peptide, 26.8 ng/mL for IGF-I, and 31.8 ng/mL for IGF-II, respectively.

*The mediation effects presented are the change (95% CI) in the outcome (birth weight or length z score) per SD increment in cord blood insulin, proinsulin, C-peptide, IGF-I or IGF-II (evaluated separately) that could account for the effect of cord blood FGF19 on fetal growth (birth weight or length). The mediation effects were estimated by the product (Baron and Kenney) method.

P values in bold: P < 0.05.

## Discussion

### Main Findings

We observed that GDM was not associated with cord blood FGF19. FGF19 was positively correlated with fetal growth (birth weight or length) in females only, suggesting a sex-dimorphic role of FGF19 in fetal growth.

### Data Interpretation and Comparisons With Findings in Previous Studies

Decreased circulating FGF19 levels have been reported in women with GDM (20), and in subjects with type 2 diabetes (33). Circulating FGF19 levels appear to be unrelated to age or sex in adults (34, 35). We are unaware of any reports on whether there are altered cord blood FGF19 concentrations in GDM. We observed similar cord plasma FGF19 concentrations in GDM and euglycemic pregnancies, and in male and female newborns, suggesting no impact of GDM or fetal sex on fetal FGF19 levels.

A previous study did not detect any association between caesarean section on cord blood FGF19, probably due to small sample size (11 caesarean, 33 vaginal deliveries) and low power (25). In contrast, our much larger study (142 caesarean, 163 vaginal deliveries) demonstrated higher cord blood FGF19 concentrations even in elective caesarean deliveries (n=92). Gestational age has been negatively correlated with neonatal blood FGF19 concentration (36). This is consistent with our observed negative correlation between gestational age and cord blood FGF19 concentration. FGF19 appears to be secreted at higher levels at earlier gestational ages.

A small study (n=44) (25) did not find any difference in cord FGF19 concentrations between birth weight small- vs. appropriate-for-gestational-age infants (no sex specific data). In contrast, we observed that cord blood FGF19 was positively correlated with birth weight and length in females only, suggesting that FGF19 may have a sex-dimorphic role in fetal growth. The observation adds to the growing evidence that there may be sex-specific impacts of certain early life factors (32). Females are more insulin resistant than males at birth (26, 37). Sex difference in fetal growth may emerge *via* sex specific intrauterine endocrine or metabolic factors (38). FGF19 may play a role in skeletal muscle growth and protein synthesis (7, 23, 24). We speculate that FGF19 may promote fetal growth in females under an unknown female fetus-specific endocrine environment.

Our data provide some weak evidence suggesting that insulin may mediate the association between FGF19 and fetal growth in females. Mediation analyses showed that cord blood proinsulin - a precursor to insulin, a surrogate indicator of insulin secretion, partly mediated the positive association between FGF19 and birth weight or length in female newborns, but no mediation effects were observed for insulin, C-peptide, IGF-I or IGF-II. A study in diabetic patients reported that serum FGF19 was positively associated with insulin (11). We did not detect any association between cord blood FGF19 and insulin. However, insulin levels may only reflect short-term glucose exposure levels. We did observe that cord plasma FGF19 was positively correlated with proinsulin and C-peptide which are indicators of chronic glucose exposure levels.

Interestingly, opposite to the positive correlations between cord plasma FGF19 and C-peptide or proinsulin in females, negative correlations were observed in males. We are unaware of any reports on the correlations between FGF19 and insulin or its secretion related C-peptide and proinsulin in newborns. Our data are suggestive of a sex dimorphic association between FGF19 and chronic insulin secretion levels during fetal life. The positive correlation between FGF19 and C-peptide is consistent with the positive correlation between FGF19 and fetal growth in females. However, the negative correlation between FGF19 and C-peptide is incongruent with the absence of a negative correlation between FGF19 and fetal growth in males, suggesting the need for caution in data interpretation. More studies are warranted to confirm this finding.

Circulating FGF19 and insulin concentrations are positively correlated in patients with type 2 diabetes (11), suggesting that FGF19 may participate in insulin-dependent glucose regulation. Individuals with isolated impaired fasting glucose have decreased serum FGF19 levels, suggesting that FGF19 may play a role in basal insulin secretion (33). These findings are consistent with the observed positive correlation between cord plasma FGF19 and C-peptide in female newborns. Unexpectedly, we observed a negative correlation between cord blood FGF19 and C-peptide in males. Further studies are warranted to verify whether this may be a true or chance finding.

### Strengths, Limitations and Future Research Directions

The main strengths include the large birth cohort, timely collection and processing of cord blood specimens, and high-quality biochemical assays (low inter-assay and intra-assay coefficients of variation). The main limitation is the observational nature of the study; causality cannot be ascertained. We had no data on inflammatory biomarkers. Future studies may explore whether fetal FGF19 levels are correlated with inflammatory biomarkers. The study was limited to Chinese subjects. More studies in other ethnic groups are required to understand the generalizability of the study findings.

In conclusion, our study data indicate that GDM may not affect cord blood FGF19 levels, and that there may be a sex-dimorphic role of FGF19 in fetal growth. The observations call for more studies to validate the novel findings and elucidate the underlying mechanisms.

## Data Availability Statement

The datasets presented in this article are not readily available because access to the deidentified participant research data must be approved by the research ethics board on a case-by-case basis, please contact the corresponding authors (zcluo@lunenfeld.ca; ouyangfengxiu@xinhuamed.com.cn) for assistance in data access request.

## Ethics Statement

The studies involving human participants were reviewed and approved by Xinhua Hospital, Shanghai Jiao-Tong University School of Medicine, reference number M2013-010. The patients/participants provided their written informed consent to participate in this study.

## Author Contributions

Z-CL, G-HZ, J-GF, FL, JL, JZ, and FO conceived the study. M-NY, RH, YJ-X, XL, W-JW, HH, FF, G-HZ, TZ, FL, JZ, JL, FO, and Z-CL contributed to the acquisition of research data. M-NY and RH conducted the literature review, data analysis, and drafted the manuscript. All authors contributed to revising the article critically for important intellectual content and approved the final version for publication.

## Funding

Supported by research grants from the Ministry of Science and Technology of China (2019YFA0802501, 2017YFE0124700), the Shanghai Municipal Health Commission (2020CXJQ01), the Shanghai Municipal Science and Technology Commission (19410713500, 21410713500), the National Natural Science Foundation of China (82073570, 81961128023, 81903323, 81761128035 and 81930095), the National Human Genetic Resources Sharing Service Platform (2005DKA21300), and the Canadian Institutes of Health Research (158616). The funders have no role in all aspects of the study, including study design, data collection and analysis, the preparation of the manuscript and the decision for publication.

## Conflict of Interest

The authors declare that the research was conducted in the absence of any commercial or financial relationships that could be construed as a potential conflict of interest.

## Publisher’s Note

All claims expressed in this article are solely those of the authors and do not necessarily represent those of their affiliated organizations, or those of the publisher, the editors and the reviewers. Any product that may be evaluated in this article, or claim that may be made by its manufacturer, is not guaranteed or endorsed by the publisher.

## Abbreviations

FGF19, fibroblast growth factor 19; GDM, gestational diabetes mellitus; OGTT, oral glucose tolerance test; SBC, Shanghai Birth Cohort; IADPSG, International Association of Diabetes and Pregnancy Study Groups; IGF-I, insulin-like growth factor I; IGF-II, insulin-like growth factor II; CI, confidence interval; SD, standard deviation.
